# Employment pathways of full and partial disability pensioners in Finland: a sequence analysis

**DOI:** 10.1186/s12889-026-27273-9

**Published:** 2026-04-06

**Authors:** Anu Polvinen

**Affiliations:** Finnish Centre for Pensions, Eläketurvakeskus, Helsinki, FI-00065 Finland

**Keywords:** Disability pension, Full disability pension, Partial disability pension, Work, Register data, Sequence analysis

## Abstract

**Background:**

Low employment rates among people with reduced work capacity pose a significant challenge for social and labor market policies in many countries. This study aims to identify employment pathways of full and partial disability retirees in Finland.

**Methods:**

Register data were collected on 17,443 Finns aged 20–62 who received an earnings-related full or partial disability pension in 2018. Participants were observed for 60 months from the start of their disability pension. Sequence analysis combined with clustering was used to identify typical employment pathways while receiving a disability pension. Furthermore, the different pathways were described according to individual-level factors.

**Results:**

Around 25% of the study population belonged to clusters where people received a partial disability pension and worked throughout their pension period. Only a small proportion received a partial disability pension and did not work. Over 60% belonged to clusters where people received a full disability pension and did not work, or worked for a brief initial period. Only very few full disability pensioners continued to work for more than 12 months. Many individual-level factors were linked with different pathways of work.

**Conclusions:**

Disability pension recipients exhibited distinct employment pathways that varied by pension type and individual-level factors. The findings highlight substantial differences in labor market participation potential, emphasizing the need for tailored policy approaches.

## Introduction

Labor participation rates among individuals with reduced work capacity remain notably low in many countries, despite continuing efforts to improve their employment prospects [1–4]. In Finland, around 5% of the working-age population receives a disability pension [5]. Although Finnish pension rules allow people to continue to work while receiving a disability pension, this remains relatively uncommon, particularly among full disability pension recipients. Nevertheless, the benefits of employment for individuals with reduced work capacity are well documented [1–4].

Under the Finnish pension system, individuals may be granted a partial or full disability pension [6]. Paid employment while drawing a partial disability pension is relatively common: over 80% continue to work while on a pension [7–9]. By contrast, only a small minority of full disability pensioners participate in paid employment [7, 9]. While we have good data on employment rates for full and partial disability pensioners, less is known about individual employment pathways among disability pensioners. A better understanding of employment periods requires more detailed information on labor force participation among individuals receiving a full or partial disability pension.

A previous Finnish study [10] examined the full-time equivalent working life expectancy (FTE-WLE) of disability pensioners at age 45, truncated at age 63, between 2005 and 2018. FTE-WLE was defined as the number of expected working years converted into full-time equivalent years. FTE-WLE for permanent full disability pensioners was 3.5 months for both men and women, rising to six months for those with musculoskeletal diseases. FTE-WLE for partial disability pensioners aged 45 was significantly longer: 8 years for women and 6.5 years for men [10]. Another Finnish study [8] reported that working throughout the entire partial disability pension period is relatively common. However, the figures from these studies are based on averages and shed no light on employment pathways at an individual level.

Previous research has identified differences in labor force participation among disability pension recipients [7, 9, 11]. Individuals who work while receiving a full or a partial disability pension tend to be more often highly educated and to have received their pension for a shorter period of time. Furthermore, full disability pensioners with mental disorders are less likely to be employed than those with other diagnoses [7, 9]. Prior employment also comes into play: individuals who have previously been employed are more likely to continue working than those who have been unemployed or outside the labor market [8, 9]. Previous Finnish studies have found evidence that working while on a disability pension is more common among public than private sector employees [9]. Employment opportunities depend in part on the types of jobs available, as physically demanding and low-skilled jobs may pose specific difficulties for individuals with limited work capacity. Despite these insights, little is known about how individual-level factors are linked with the various employment pathways of disability pensioners.

Increasing the employment rate in the working-age population is a key policy objective in many countries [1–3]. Achieving this goal requires an improved understanding of labor market participation among individuals with reduced work capacity. This study examines the employment pathways of individuals who began receiving full or partial disability pensions in 2018 by tracking their labor market participation over a five-year period.

### Disability pensions in Finland

In Finland, individuals with an illness, disability or injury that reduces their ability to work for at least 12 months may be eligible for a disability pension. Typically, the pension is preceded by a period of sickness allowance. Prospects of maintaining or restoring the individual’s work ability are assessed based on appropriate medical treatment, rehabilitation and workplace adjustments. These are essential steps before decision-making on the pension. If the individual’s work ability does not improve sufficiently, they may apply for a disability pension. A full disability pension may be granted if their work capacity is reduced by at least three-fifths, while a partial disability pension is granted if it is reduced by at least two-fifths. Both types of disability pension can be granted either temporarily or until further notice. A temporary disability pension is granted if it is thought that it might be possible to restore the person’s work ability through rehabilitation. Currently, more than half of all disability pensions are granted on a temporary basis. However, the majority of these later become permanent [12].

In recent years, nearly 30% of all new disability pensions granted in Finland have been partial pensions. A partial disability pension is half the value of the corresponding full pension. While full disability pensions are intended for people with significantly impaired work ability, individuals who are granted a partial disability pension are encouraged to continue working within their remaining capabilities. Those receiving a full disability pension may earn up to 40% of their pre-disability income. For those on a partial disability pension, the earnings limit is set at 60%. However, if an individual’s earnings prior to receiving a pension were relatively low, they may earn a fixed monthly amount of €986.30 (in 2025) without exceeding the threshold [12].

The Finnish pension system has a flexible retirement age, which allows people to retire at any time after reaching the minimum age for claiming an old-age pension. However, when disability pension recipients reach the minimum retirement age, their disability pension is automatically converted into an old-age pension. As part of the 2017 pension reform, it was decided that this minimum age would be incrementally raised from 63 to 65 [6, 12].

## Methods

The administrative registers of the Finnish Centre for Pensions and Statistics Finland provided complete data with only minor missing entries. Data were collected on all Finnish individuals aged 20–62 who began receiving an earnings-related partial or full disability pension in 2018 (*N* = 17,443). The register data included exact information on disability pension periods as well as the reason for the pension. Additionally, the dataset contained monthly information on days in employment as well as information on the gender, age, education and employment sector of study participants prior to disability retirement.

### Monthly statuses

Individuals receiving a disability pension may choose to either continue working or not work at all during the pension period. The register data included the exact start and end dates of employment and disability pension (including full and partial pensions) between 2018 and 2023. However, they did not detail whether employment was part-time or full-time. Based on this employment and disability pension information, monthly statuses were compiled for all disability pensioners. These individuals were observed for 60 months, starting from the beginning of the month in which their disability pension began in 2018.

Five mutually exclusive states were created for each person over the 60-month follow-up period: (1) receiving a full disability pension and working; (2) receiving a full disability pension and not working; (3) receiving a partial disability pension and working; (4) receiving a partial disability pension and not working; and (5) not receiving a full or a partial disability pension. Disability pension periods were defined to monthly accuracy, and recipients of disability pensions were classified as working if they had worked for at least ten days in that month. If a person was not receiving a full or a partial disability pension, this indicated that they were either employed, unemployed, deceased, retired due to old age, or otherwise inactive.

### Background variables

In 2018, age was classified into the following categories: 20–39, 40–49, 50–59, and 60–62 years. Highest level of education was classified as (1) primary school (up to 9 years of education), (2) secondary school (up to 12 years of education), or (3) lower (up to 15 years of education) or higher tertiary education (16 + years of education).

Information on individuals’ employment sector prior to retirement on a disability pension was based on information from 2016. Employment status two years prior to the onset of disability was categorized as follows: (1) employee in the private sector; (2) employee in the public sector; (3) self-employed; (4) not working.

Information on disability pension diagnoses and on whether the pension was granted temporarily or until further notice was obtained from retirement data. The diagnoses were categorized into two ICD-10 groups: mental and behavioral disorders (F00–F99) and somatic diseases (covering all other ICD-10 diagnostic categories beyond F00–F99).

Sequence and cluster analysis.

Sequences were created for the study population using monthly statuses, ensuring that each individual had a 60-month sequence starting from the date they began receiving a disability pension. Sequence and cluster analysis were then used to analyze the sequences and to identify typical patterns for disability retirees. Optimal matching techniques were used to quantify the similarities and distances between pairs of sequences. Transition rates between states were used to define substitution costs: a substitution cost between two states is lower if transitions between those states are more common. Transition-rate based substitution costs are commonly used as a data-driven method of reducing bias arising from arbitrary or uniform cost settings. The indel cost was set to the default value 1. The Ward method was used for clustering. Analyses were performed using the TraMineR and WeightedCluster packages [13–15].

An optimal cluster solution was found by comparing the performance of various solutions based on Average Silhouette Width (ASW) values for cluster quality, and an assessment of whether the clusters could be meaningfully distinguished from one another. The seven-cluster solution was selected because it produced the best ASW value (0.4420). The PBC value (0.6388) was also high in the seven-cluster solution.

## Results

**Fig. 1:**
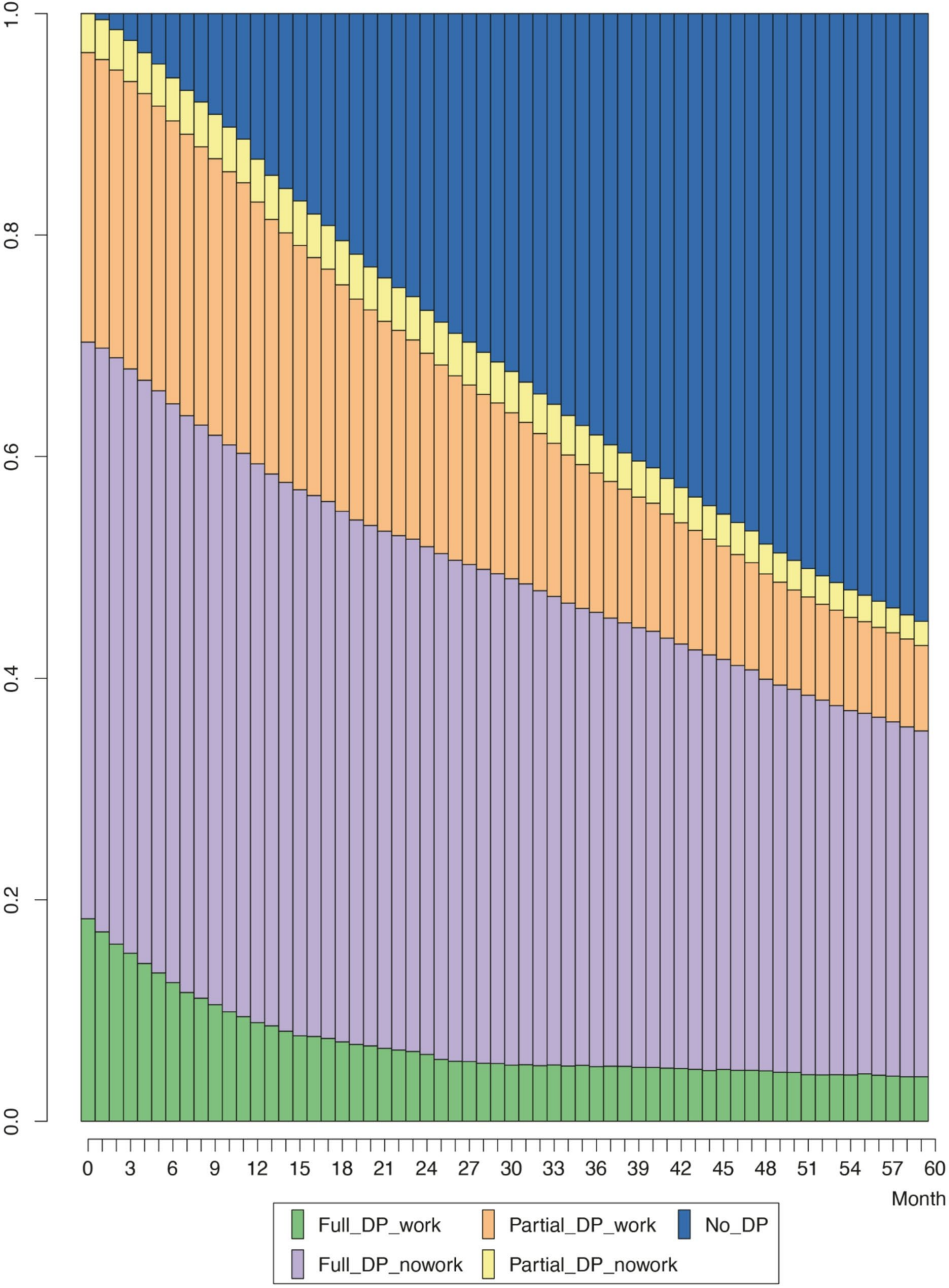
Monthly statuses for 60 months after entering disability pension (DP)

The distribution plot in Fig. 1 illustrates the frequency of different states over time. The results show that 26% started receiving a partial disability pension and were working. Only very few received a partial disability pension and did not work (4%). Over half or 52% began receiving a full disability pension and did not work while drawing their pension. Additionally, 18% received a full disability pension and continued to work.

The proportion of people receiving full or partial disability pensions began to decrease over time. By the end of the follow-up period, 45% of study participants were still receiving a disability pension; 8% received a partial disability pension and were working; and 2% received a partial disability pension and were not working. 4% received a full disability pension and were working, while 31% received a full disability pension and were not working.

**Fig. 2:**
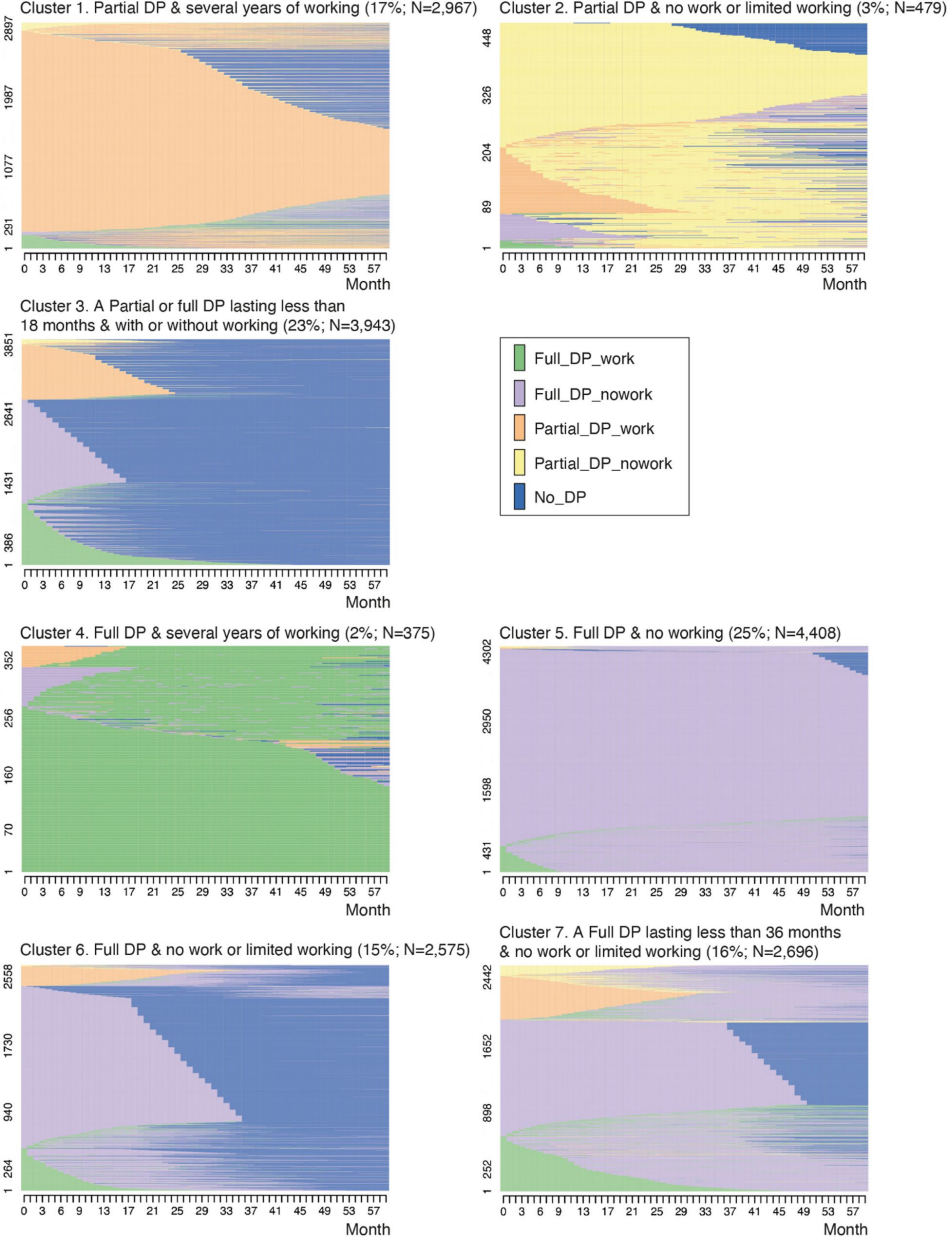
Individual sequences 60 months after entering a disability pension (DP)

Optimal matching techniques and cluster analysis yielded a seven-cluster solution (Fig. 2). Approximately 17% of the study population belonged to the cluster where individuals received a partial disability pension for several years while working simultaneously (cluster 1). 3% belonged to cluster 2, which comprised individuals who received a partial disability pension for several years. Most of these individuals did not work, although some did work for a brief initial period. Cluster 3 (23%) included those who received a partial or full disability pension for a shorter period, during which they either worked or did not work. Almost all partial disability pensioners in this cluster worked throughout the entire pension period.

Only very few people were in paid employment while drawing a full disability pension. 2% of the study population belonged to cluster 4, which comprised individuals who received a full disability pension for several years while continuing to work throughout this period. The majority (56%) belonged to clusters 5–7, where individuals received a full disability pension for either the entire period (cluster 5) or a slightly shorter period (clusters 6 and 7) and did not work. However, a small proportion initially combined a disability pension with employment but stopped working after a few months.

Table 1 shows the descriptive statistics for each cluster. The results show that recipients of a partial disability pension (cluster 1) who continued to work were often women, typically over 50 years of age, and highly educated. Over 50% of them had worked in the public sector, and only very few had been outside the labor market before their disability retirement. The majority had retired due to somatic diseases.

**Table 1:** Descriptive statistics of clusters*^)^

		Cluster 1. Partial DP & several years of working (17%; *N* = 2,967)	Cluster 2 Partial DP & no working or limited working (3%; *N* = 479)	Cluster 3. Partial or full DP lasting less than 18 months & with or without working (23%; *N* = 3,943)	Cluster 4. Full DP & several years of working (2%; *N* = 375)	Cluster 5. Full DP & no working (25%; *N* = 4,408)	Cluster 6. Full DP & no work or limited working (15%; *N* = 2,575)	Cluster 7. Full DP lasting less than 36 months & no work or limited working (16%; *N* = 2,696)	All (100%; *N* = 17,443)
Gender	Women	73.0 (*p* < 0.0001)	53.9 (*p* = 0.5617)	57.4 (*p* = 0.0014)	66.1 (*p* < 0.0001)	45.4 (*p* < 0.0001)	52.9 (*p* = 0.0121)	49.2 (*p* < 0.0001)	55.2
Age	20–39	4.5 (*p* < 0.0001)	4.8 (*p* < 0.0001)	22.1 (*p* < 0.0001)	17.0 (*p* < 0.0001)	19.9 (*p* < 0.0001)	12.9 (*p* < 0.0001)	15.7 (*p* < 0.0001)	15.7
	40–49	11.2	10.0	19.6	24.5	17.5	9.8	9.4	14.4
	50–59	56.1	66.4	28.4	53.1	58.1	39.7	16.1	42.0
	60–62	28.1	18.8	30.0	5.3	4.6	37.6	58.8	28.0
Education	Primary	8.9 (*p* < 0.0001)	18.6 (*p* = 0.9521)	18.5 (*p* = 0.4460)	9.3 (*p* < 0.0001)	20.6 (*p* < 0.0001)	19.6 (*p* = 0.0850)	24.0 (*p* < 0.0001)	18.2
	Secondary	56.1	56.2	56.0	51.2	59.1	56.6	56.4	56.9
	Tertiary	35.0	25.3	25.5	39.5	20.4	23.8	19.6	24.9
Employment sector two years prior to disability pension	Private	32.8 (*p* < 0.0001)	32.6 (*p* < 0.0001)	40.7 (*p* < 0.0001)	23.2 (*p* < 0.0001)	29.1 (*p* < 0.0001)	34.9 (*p* = 0.3984)	36.3 (*p* < 0.0001)	34.3
	Public	58.1	15.9	28.7	55.5	8.0	25.2	17.9	26.5
	Self-employed	6.9	11.7	6.6	16.3	6.8	7.1	7.1	7.2
	None	2.2	39.9	24.0	5.1	56.1	32.9	38.8	32.0
Diagnosis	Mental	21.1 (*p* < 0.0001)	14.6 (*p* < 0.0001)	27.6 (*p* < 0.0001)	44.8 (*p* < 0.0001)	47.5 (*p* < 0.0001)	31.5 (*p* = 0.6051)	26.7 (*p* < 0.0001)	32.0
	Somatic	78.9	85.4	72.4	55.2	52.5	68.5	73.3	68.0
DP granted as temporary	Yes	47.5 (*p* < 0.0001)	34.4 (*p* < 0.0001)	71.9 (*p* < 0.0001)	82.7 (*p* < 0.0001)	65.5 (*p* < 0.0001)	66.6 (*p* < 0.0001)	47.6 (*p* < 0.0001)	58.7
Deceased over follow-up	Yes	1.6 (*p* < 0.0001)	2.5 (*p* < 0.0001)	13.4 (*p* < 0.0001)	2.4 (*p* < 0.0001)	3.1 (*p* < 0.0001)	9.1 (*p* = 0.0490)	16.8 (*p* < 0.0001)	8.2

*^)^ Values are shown as percentages in the columns and cluster sizes (N&%) are shown in the column headers

The statistical significance of each cluster compared with other clusters is based on a Chi-squared test (*p*-values)

Those receiving a partial disability pension but not continuing to work (cluster 2) were most often aged 50–59 and had completed secondary education. 40% had been outside the labor market before their disability retirement. Their pensions were often granted due to somatic diseases. The proportion of men was higher in this cluster than among partial disability pensioners who continued working (cluster 1).

Cluster 3 included those partial or full disability recipients who had received a pension for less than 18 months. Over half of them were in paid employment while drawing a partial or full disability pension. Most of them had worked in the private sector and over 70% had retired on a disability pension due to somatic diseases. Their pensions were quite often granted on a temporary basis. 13% had died during the follow-up period.

In cluster 4, where individuals worked while receiving a full disability pension, the majority were women and 80% were aged 40–59 years. Most of them had been employed before their disability retirement. Over half had worked in the public sector and a considerable number had been in self-employment. Their pension was often granted on a temporary basis.

Those in cluster 5, who experienced a longer period of full disability pension and were not in paid employment, were more often men than women, younger than average, and less educated. Almost 60% of them had been outside of the labor market prior to their disability retirement. In this cluster, many (48%) had disability retired due to mental disorders.

Those who received a full disability pension for a shorter period and were not working or who worked for a limited period (clusters 6 and 7) were older than average and relatively often men. Almost 60% of individuals in cluster 7 were aged 60–62. Many of their disability pensions were due to somatic diseases. Almost one-fifth of them died over the study period.

## Discussion

This study used sequence analysis and clustering techniques to identify employment pathways among Finnish recipients of full and partial disability pensions. The analysis revealed several distinct trajectories combining employment and pension receipt. These pathways varied according to pension type (partial or full) and outcome, including sustained employment over several years, limited employment, no employment, and other combinations. Furthermore, specific combinations of pension type and employment status varied according to certain individual-level characteristics.

Working while receiving a partial disability pension was relatively common, whereas employment during a full disability pension was much less so. Many partial disability pensioners continued to work throughout their pension period, whereas this was relatively uncommon among full disability pensioners. This difference may partly be explained by a more severely reduced work capacity among full disability pensioners, which may well hamper long-term employment. In contrast, individuals receiving a partial disability pension typically have better work capacity, offering better opportunities to remain in the workforce [6].

Full disability pension recipients who continued to work for several years while drawing their pension were relatively similar to partial disability pension recipients in terms of their individual characteristics. This is in line with previous results which have found that working while receiving a disability pension is more common among women, the higher-educated, public sector employees, and those who had been employed prior to disability retirement [7–9, 11]. This may be because individuals with higher education and stable career paths are better placed to continue working part-time while drawing a pension. People with higher education are less likely to work in physically strenuous conditions than those with a lower level of education, and they have better prospects of remaining employed for longer [16, 17]. Furthermore, earlier research on workplace differences [18, 19] has suggested that larger employers are better equipped to accommodate part-time working arrangements than smaller ones. Since public sector workplaces are usually large, this may partly explain why working alongside a disability pension is more common in the public sector than in the private sector.

Most full disability pension recipients did not participate in paid employment during their pension period. Those whose disability pension began as a full disability pension and who were not working at the start of their disability retirement were less likely to start working later while receiving their disability pension. Those full disability pensioners who remained outside the labor market were more often men, younger than average, and with lower levels of education. They had relatively often been disability retired due to mental disorders and outside the labor market prior to disability retirement. These characteristics are known to be associated with weaker labor market attachment [20–25]. A previous study [7] has also found a lower employment rate among young full disability pension recipients, which may be partly due to poorer health or reduced work capacity, limited labor market experience, and insufficient skills or qualifications. Furthermore, most disability pensions granted to young individuals are due to mental disorders [26]. These findings highlight the importance of targeted interventions for people diagnosed with mental disorders.

Among disability pension recipients who were not employed, a small subgroup participated in short-term work for a few months after their disability retirement. Some of these individuals received a partial disability pension before transitioning to a full disability pension. Their employment may have ended for various reasons, such as deteriorating health, reduced work capacity, or workplace-related conditions or circumstances. Previous studies [27–29] have shown that a relatively high proportion of partial disability pension recipients transition to a full disability pension within a few years. This shift is particularly common among men, older individuals, individuals with lower educational attainment, and those whose partial disability pension was granted on a temporary basis or due to mental disorders. Further research should more closely examine the barriers to employment and the reasons why individuals stop working while receiving a disability pension.

During the study period, some disability pension recipients reached retirement age and transferred to an old-age pension. This was likely most common in clusters 3, 6 and 7, which comprised the oldest disability pensioners. A disability pension may also end for other reasons. Most disability pensions that are initially granted on a temporary basis, become permanent within a few years, although some pension recipients do return to work or become unemployed [30]. 8% of the study participants died during the study period.

This study identified several different employment pathways for people who had retired on a disability pension. These pathways varied according to individual-level factors. These findings are highly relevant for public health and labor market policymaking and highlight the need for a differentiated rather than uniform approach to disability pension recipients.

Supporting the employment of people with partial work capacity can help to maintain their ability to work, their social participation, and their economic security. All these factors are key determinants of health and wellbeing. However, effective targeting of such support requires recognition that work capacity among disability pension recipients can vary substantially. The results suggest that partial disability pensions can facilitate continued participation in the labor market among individuals with reduced work capacity. At the same time, the low level of employment among recipients of full disability pensions indicates that expectations regarding work participation should be aligned with individuals’ health-related limitations. Overall, the findings emphasize the importance of coordinated public health, social security, and labor market policies that consider differing work capacities.

### Limitations of the study

There is limited research on employment among disability pensioners and existing studies are outdated. This study sheds new light on the employment patterns of both partial and full disability pension recipients. High-quality data allowed us to provide monthly status information for each individual, including details on the receipt of a full or partial disability pension, as well as periods of employment. The follow-up period spanned 60 months from the start of disability pension. Sequence and cluster analysis were applied to identify different employment pathways among individuals whose disability pension began in 2018. The ASW and PBC values were good, meaning that the cluster solution successfully distinguished different patterns of employment among full and partial disability pension recipients over the five-year study period.

While the study has several methodological strengths, it does have some limitations that should be acknowledged. Firstly, the study period included the COVID-19 pandemic, which may have had a bearing on the results. Secondly, the descriptive design restricted the ability to infer causal relationships between individual-level factors and different employment pathways. Thirdly, the available data lacked detailed information on working conditions. And fourthly, the definition of employment applied — having worked for at least 10 days within a given month — is somewhat arbitrary and may not have fully captured the complexity of irregular or marginal employment patterns. These limitations highlight the need for future research that incorporates richer longitudinal data, more precise measures of work characteristics and alternative definitions of employment. Further studies could also build on our findings by using causal modelling approaches to explore the underlying mechanisms of the observed associations.

## Conclusions

Disability pensioners are a diverse group with widely varying employment pathways. Employment is common and often sustained among partial disability pension recipients, whereas work participation among those receiving a full disability pension is rare and usually short-lived. These findings are relevant to public health and labor market policymaking, as they highlight significant variations in remaining work capacity and thus emphasize the need for tailored approaches to support work participation among disability pension recipients.

## Data Availability

The author used individual-level register data from the Finnish Centre for Pensions and Statistics Finland. However, due to legal restrictions and data protection regulations, the author is not permitted to make this sensitive personal data available.

## Abbreviations

DP: Disability Pension
FTE-WLE: Full-Time Equivalent Working Life Expectancy
ICD-10: The International Classification of Diseases
F00–F99: Mental and behavioral disorders
ASW: Average Silhouette Width
PBC: Point Biserial Correlation
